# Targeting transcription factor pu.1 for improving neurologic outcomes after spinal cord injury

**DOI:** 10.3389/fnins.2024.1418615

**Published:** 2024-08-15

**Authors:** Yi Shi, Meige Zheng, Yang Luo, Jianjian Li, Fangru Ouyang, Yuanzhe Zhao, Jingwen Wang, Zhida Ma, Congpeng Meng, Yihui Bi, Li Cheng, Juehua Jing

**Affiliations:** ^1^Department of Orthopedics, The Second Affiliated Hospital of Anhui Medical University, Hefei, China; ^2^Institute of Orthopedics, Research Center for Translational Medicine, The Second Affiliated Hospital of Anhui Medical University, Hefei, China

**Keywords:** spinal cord injury, pu.1, lipid droplet, cholesterol crystals, lipid metabolism

## Abstract

**Background:**

After spinal cord injury (SCI), lipid metabolism dysregulation at the lesion site exacerbates secondary damage. The transcription factor pu.1 has been implicated as a negative regulator of multiple lipid metabolism-related genes and pathways. However, its role in post-SCI lipid metabolism remains unclear.

**Methods:**

We employed a mouse model of complete T10 crush SCI. Non-targeted metabolomics and bioinformatics analysis were utilized to investigate lipid metabolism at the lesion site after SCI. Polarized light imaging was used to evaluate the presence of cholesterol crystals. DB1976, a specific inhibitor of pu.1, was administered to examine its impact on local lipid metabolism after SCI. Immunofluorescence staining was performed to assess pu.1 expression and distribution, and to evaluate lipid droplet formation, astrocytic/fibrotic scar development, inflammatory cell infiltration, and tight junctions within the vasculature.

**Results:**

Non-targeted metabolomics and bioinformatics analyses revealed significant alterations in lipid metabolism components after SCI. Moreover, immunofluorescence staining and polarized light imaging demonstrated substantial BODIPY^+^ lipid droplet accumulation and persistent cholesterol crystal formation at the lesion site after SCI. Increased pu.1 expression was predominantly observed within macrophages/microglia at the lesion site after SCI. DB1976 treatment significantly mitigated lipid droplet accumulation and cholesterol crystal formation, reduced CD68^+^ macrophage/microglial infiltration, and attenuated fibrotic scar formation. Moreover, DB1976 treatment promoted the expression of claudin-5 and zonula occludens-1 between vascular endothelial cells and enhanced GFAP^+^ glial connectivity after SCI.

**Conclusion:**

Our study reveals a significant correlation between lipid metabolism disturbance post-SCI and transcription factor pu.1 upregulation, specifically in macrophages/microglia at the lesion site. Thus, targeted pu.1 modulation has the potential to yield promising results by substantially diminishing the deposition of lipid metabolism byproducts at the lesion site and fostering a milieu conducive to SCI repair.

## 1 Introduction

Myelin contains a high proportion of lipids, with approximately 70–75% of its dry weight comprising cholesterol, phospholipids, and glycolipids (Chrast et al., 2011). Spinal cord injury (SCI) results in extensive myelin debris accumulation, creating a distinctive lipid-dense environment (Wang et al., 2015; Kopper and Gensel, 2018; Zheng et al., 2023). While myelin debris clearance is predominantly orchestrated by activated macrophages/microglia, excessive lipid uptake triggers the formation of lipid-laden macrophages, commonly referred to as foamy cells (Greenhalgh and David, 2014; Wang et al., 2015, 2024; Kopper and Gensel, 2018; Ryan et al., 2022), which can persist at the lesion site for up to a year post-SCI (Fleming et al., 2006). Foamy macrophages contribute to prolonged inflammation, exacerbating gliosis, fibrosis, and neuronal cell death at the lesion site, thereby establishing an unfavorable tissue microenvironment for regeneration (Ren and Young, 2013; Gensel and Zhang, 2015; Milich et al., 2019; Wang et al., 2024).

Stimulating lipid efflux with AdipoRon, an agonist for the atheroprotective hormone adiponectin, has been shown to reduce lipid accumulation at the lesion site, alleviate post-SCI inflammation, and restore motor function (Zhou et al., 2019). Genetic deletion of the scavenger receptors CD36 and MSR1 have been implicated in foamy macrophage formation in atherosclerosis, leading to decreased lipid droplet numbers, reduced lesion size, and improved locomotion scores after SCI (Myers et al., 2014; Zhu et al., 2017; Kong et al., 2020). These studies underscore the potential of targeting foamy cells for therapeutic intervention in the context of SCI. Compared to non-lipid-laden macrophages, foamy cells exhibit distinct transcriptomic features (Zhu et al., 2017), suggesting that lipid deposition in macrophages is a significant cause or consequence of extensive transcriptional regulation. While lipid metabolism plays an important role in SCI, the underlying molecular links and regulatory mechanisms remain unclear.

Pu.1, encoded by the gene *Spi1*, belongs to the ETS-family of transcription factors. Recent studies have demonstrated that targeting pu.1 using small hairpin RNA (shRNA) or small-molecule inhibitors, such as DB1976, in hepatic macrophages yields promising outcomes, including liver glucose homeostasis improvement, lipid deposition reduction, and liver inflammation and fibrosis alleviation (Liu et al., 2020). In obesity, pu.1 has been identified as a negative regulator of peroxisome proliferator activated receptor gamma activity, thereby influencing adipocyte metabolism (Lackey et al., 2019). In Alzheimer’s disease, pu.1 knock-down has been shown to upregulate the expression of genes and activate pathways associated with lipid metabolism, including *APOE, ABCA1, TREM2* and *LXR/RXR* signaling, thereby facilitating lipid and debris clearance (Pimenova et al., 2021).

After SCI, persistent inflammation, blood-spinal cord barrier (BSCB) leakage, and scar formation at the lesion site pose significant pathological challenges that impede axonal regeneration and functional recovery (Hara et al., 2017; Dias and Göritz, 2018; Tran et al., 2018; Alizadeh et al., 2019; Kobayakawa et al., 2019). However, the intricate mechanisms underlying these pathological changes have yet to be elucidated. Moreover, while pu.1 has emerged as a pivotal regulator in metabolic diseases, its specific role in governing lipid metabolism after SCI remains elusive. Therefore, this study aimed to determine the role of lipid metabolism in SCI, specifically the impact of the transcription factor pu.1.

## 2 Materials and methods

### 2.1 Animals and SCI model

All animal experiments were conducted in accordance with the guidelines and regulations of the Animal Ethics Committee of Anhui Medical University (approval no. LLSC20160052). Female C57BL/6J mice aged 8–10 weeks old and weighing 20–22 g were purchased from the Animal Experiment Center of Anhui Medical University. All mice were housed in a temperature-controlled environment under a 12 h light-dark cycle and provided with *ad libitum* access to food and water at the Laboratory Animal Center of The Second Affiliated Hospital of Anhui Medical University.

The mice were anesthetized via intraperitoneal injection of 50 mg/kg pentobarbital sodium. Following laminectomy, the T10 segment of the spinal cord was exposed. A complete SCI mice model was established by clamping the spinal cord at the T10 level using calibrated Dumont No. 5 forceps (11252-20, Fine Science Tools, Germany) for 5 s from two sides. In the sham group, the mice underwent laminectomy without spinal cord clamping. The incisions were closed carefully layer by layer. Anti-infection and auxiliary urination nursing were performed twice a day after SCI.

### 2.2 Intrathecal DB1976 injection

DB1976 (GC39628, Glpbio, United States) was diluted with phosphate buffered saline (PBS) to a concentration of 1 μg/μl. For the DB1976 treatment group (*n* = 3 mice), immediately after SCI induction in mice, 10 μl of diluted DB1976 was intrathecally injected using a micro-syringe (1701, Hamilton, United States). The insertion site was in the dorsal midline of the L5–6 intervertebral space, as previously reported (Li et al., 2019). Successful insertion into the intrathecal space was confirmed by tail-flick observation. The control group (*n* = 3 mice) received an equal volume of PBS. Injections were administered once daily at the same time point until the day of sample collection.

### 2.3 Non-targeted metabolomics and bioinformatics analyses

To explore metabolite differences among the different groups, eighteen samples (1 cm long spinal cord segments centered on the lesion core per sample) were selected for metabolomic analysis, divided into three groups with each group containing six samples: control (pre-injury), 1 day post-injury (dpi), and 28 dpi. Samples were thawed on ice, and 50 mg of tissue from each sample was homogenized with 500 μl of ice-cold methanol/water solution (70%, v/v). After homogenization at 30 Hz for 2 min, the mixture was shaken for 5 min and incubated on ice for 15 min. Subsequently, the mixture was centrifuged at 12,000 rpm at 4°C for 10 min, and 400 μl of supernatant was transferred into another centrifuge tube. Ethyl acetate/methanol solution (v, 1:3) (500 μl) was added to the original centrifuge tube, and the mixture was oscillated for 5 min and incubated on ice for 15 min. Subsequently, the mixture was centrifuged at 12,000 rpm at 4°C for 10 min, and 400 μl of supernatant was collected. The two supernatant collections were then combined and concentrated. Then, 100 μl of 70% methanol water was added to the dried product, and ultrasonic treatment was performed for 3 min. Finally, the mixture was centrifuged at 12,000 rpm at 4°C for 3 min, and 60 μl of supernatant was extracted for liquid chromatography with tandem mass spectrometry (LC-MS/MS) analysis. All samples were processed according to the established protocols for the LC-MS system. The analytical conditions were optimized as follows: ultra-performance liquid chromatography (UPLC) columns, Waters ACQUITY UPLC HSS T3 C18 (1.8 μm, 2.1 mm × 100 mm); column temperature, 40°C; flow rate, 0.4 ml/min; injection volume, 2 μl; solvent system, water (0.1% formic acid): acetonitrile (0.1% formic acid); gradient program, 95:5 v/v at 0 min, 10:90 v/v at 11.0 min, 10:90 v/v at 12.0 min, 95:5 v/v at 12.1 min, and 95:5 v/v at 14.0 min.

The mzML format was initially generated for the original data file obtained by LC-MS analysis using ProteoWizard software. Peak extraction, alignment, and retention time correction were performed using the XCMS program. The peak area was corrected using the “SVR” method. The peaks with a deletion rate >50% in each group of samples were filtered. Subsequently, metabolic identification information was obtained by searching the laboratory’s self-built database (Wuhan Maiteweier Biotechnology Co., Ltd., China), integrating the public database Metlin,^1^ HMDB^2^ KEGG,^3^ Mona,^4^ MassBank,^5^ and metDNA.

Differential Metabolite Screening: Based on the orthogonal partial least squares discriminant analyses (OPLS-DA) results, we could preliminarily screen for differential metabolites between groups using the Variable Importance in Projection (VIP) scores obtained from the multivariate analysis OPLS-DA model. To further refine the selection of differential metabolites, we combined these results with univariate analysis metrics, including the P-values and fold change (FC). For comparisons without biological replicates, differential screening was conducted based solely on the FC values. For comparisons with biological replicates, we integrated the FC values, P-values, and VIP values from the OPLS-DA model to identify differential metabolites. The specific screening criteria were as follows:

1. Metabolites with VIP scores ≥ 1 were selected based on the OPLS-DA model; the VIP score indicates the influence of the corresponding metabolite on the classification of samples between groups within the model. Metabolites with VIP scores ≥ 1 were considered to have significant differences.

2. In case of biological replicates in the sample groups, differential metabolites were screened by incorporating the P-values from a univariate t-test, with a threshold set at *P*-values < 0.05.

3. Metabolites that showed a difference of at least 2-fold increase or 0.5-fold decrease between the control and experimental groups were selected, i.e., metabolites with a FC ≥ 2 or ≤0.5.

### 2.4 Western blot analysis

To explore the temporal expression of pu.1 before and after SCI, eighteen samples (1 cm long spinal cord segments centered on the lesion core per sample) were selected for Western blot analysis, divided into six groups with each group containing three samples: pre, 1, 3, 7, 14, and 28 dpi. For Western blot analysis, each tissue sample was homogenized in 500 μl RIPA buffer (P0013B, Beyotime, China) supplemented with 5 μl phenylmethanesulfonyl fluoride (BL615A, Biosharp, China) and a 5 μl mixture of protease inhibitor cocktails (BL612A, Biosharp, China). After 10 min of incubation on ice, the homogenates were centrifuged at 12,000 rpm for 30 min at 4°C and quantitated using a BCA quantification kit (BL521A, Biosharp, China). Protein (25 μg) from each sample was loaded onto a 10% sodium dodecyl-sulfate polyacrylamide gel electrophoresis (SDS-PAGE) gel and then transferred to nitrocellulose membranes. The membranes were blocked with 5% non-fat milk for 2 h and then probed with the following primary antibodies overnight at 4°C: rabbit anti-pu.1 (1:1000, ab227835, Abcam, United States) and mouse anti-β-actin (1:1000, 3700s, Cell Signaling Technology, United States). The membranes were incubated with the corresponding horseradish peroxidase-conjugated secondary antibodies (1:10000, A4416, A0545, Sigma-Aldrich, United States). The protein signals were visualized using the Tanon system (5200, Tanon, China) and quantified using ImageJ/Fiji software.

### 2.5 Immunofluorescence staining

For immunofluorescence staining, 3 samples from each group and 3 sections from each sample were selected. After anesthetization with pentobarbital sodium, thoracotomy was performed. After cardiac perfusion with cold PBS and 4% paraformaldehyde (Servicebio, China), the SCI tissue containing the lesion epicenter (8 mm) was removed and fixed in 4% PFA for 24 h, immersed in 30% sucrose for 72 h, and embedded in tissue freezing media (Sakura, United States). Sagittal sections (16 μm) were prepared on a cryostat (NX50, Thermo Fisher, United States), washed with PBS three times, and blocked in PBS with 10% donkey serum albumin (DSA, SL050, Solarbio, China) and 0.3% Triton X-100 (T8200, Solarbio, China) for 1 h at room temperature. Subsequently, the tissue sections were incubated at 4°C overnight with the following primary antibodies: goat anti-CD31 (1:200, AF3628, R&D Systems, United States), mouse anti-claudin-5 (CLN-5, 1:100, 35-2500, Invitrogen, United States), rabbit anti-ZO-1 (1:100, 61-7300, Invitrogen, United States), goat anti-platelet derived growth factor receptor β (PDGFRβ, 1:100, AF1042, R&D Systems, United States), rat anti-GFAP (1:200, 13-0300, Invitrogen, United States), rabbit anti-pu.1 (1:500, ab227835, Abcam, United States), rat anti-CD68 (1:500, MCA1957, AbD Serotec, United Kingdom), and rabbit anti-fibronectin (1:100, 15613-1-AP, Proteintech, China). The sections were then incubated at 37°C in the dark for 1 h with the following secondary antibodies: donkey anti-rabbit Alexa Fluor 488, donkey anti-mouse Alexa Fluor 488, donkey anti-goat Alexa Fluor 594, and donkey anti-rat Alexa Fluor 488 (1:500, A-21206, A-21202, A-21209, A-11058, Invitrogen, United States). Finally, the sections were sealed with an anti-fluorescence quencher (P0126, Beyotime Biotechnology, China).

For lipid droplet measurement, the BODIPY antibody (1 mg/ml; D3922, Invitrogen) was diluted in PBS and applied to the spinal cord sections for 30 min, followed by three 10-min PBS washes. All sections were mounted using Vectashield with DAPI (H-1200, Vector Laboratories) and covered with glass coverslips. Immunofluorescence images were obtained using an Olympus FV3000 confocal microscope and a fluorescence microscope (Axio Scope A1, Zeiss, Germany), with consistent light intensity and filtering.

### 2.6 Quantification

All images were measured using ImageJ/Fiji software. During preparation of the sagittal tissue sections, each section was numbered in order, and sections of comparable position/level from different mice were selected. For each sample, sections spanning the injured core and two adjacent sections spaced 160 μm apart were quantified (three sagittal sections per mice were immunostained) and the results from each section were averaged, with three samples per group. The image shown in the figures represent one of these stained sections. For quantification of CD68^+^, Iba1^+^, Mac-2^+^, PDGFRβ^+^, GFAP^+^, CD31^+^, and pu.1^+^ cells, 100 μm square grids were generated over the lesion site. Every sixth square was quantified, and only DAPI^+^ cells were counted. Images of the lesion epicenter taken for each sample were used for quantification. For BODIPY^+^, Crystal^+^, CD68^+^, PDGFRβ^+^, fibronectin^+^, collagen 1^+^, and GFAP^–^ quantification (%) in the injured spinal cord sections, the average value of each section was normalized to the area of the spinal cord segment spanning the lesion site in a 10 × image. The spinal cord lesion site was defined as the GFAP^–^ region.

### 2.7 Statistical analysis

All measurements, analyses, and statistics were performed in a blinded manner. Data are expressed as the mean ± standard error of mean (SEM). Statistical analyses were performed using Prism 9.0 software (GraphPad, United States). Significance was determined using two-tailed Student’s *t*-tests to compare two groups, while one- or two-way analysis of variance (ANOVA) with a *post hoc* Tukey–Kramer test were for multiple group comparisons. The metabolomics and bioinformatics analyses were conducted using R software (R Foundation for Statistical Computing, Vienna, Austria): principal component (PCA) and Volcano plot: R (base package) 3.5.0 version, orthogonal partial least squares discriminant analyses (OPLS-DA): MetaboAnalystR (R) 1.0.1 version, Heatmaps: pheatmap (R) 1.0.12 version. Student’s *t*-test and variance multiple analysis were employed for univariate statistical analysis, while PCA and OPLS-DA were utilized for multivariate statistical analysis. *P*-values < 0.05 were considered statistically significant.

## 3 Results

### 3.1 Local lipid metabolism dysfunction in SCI

**FIGURE 1:**
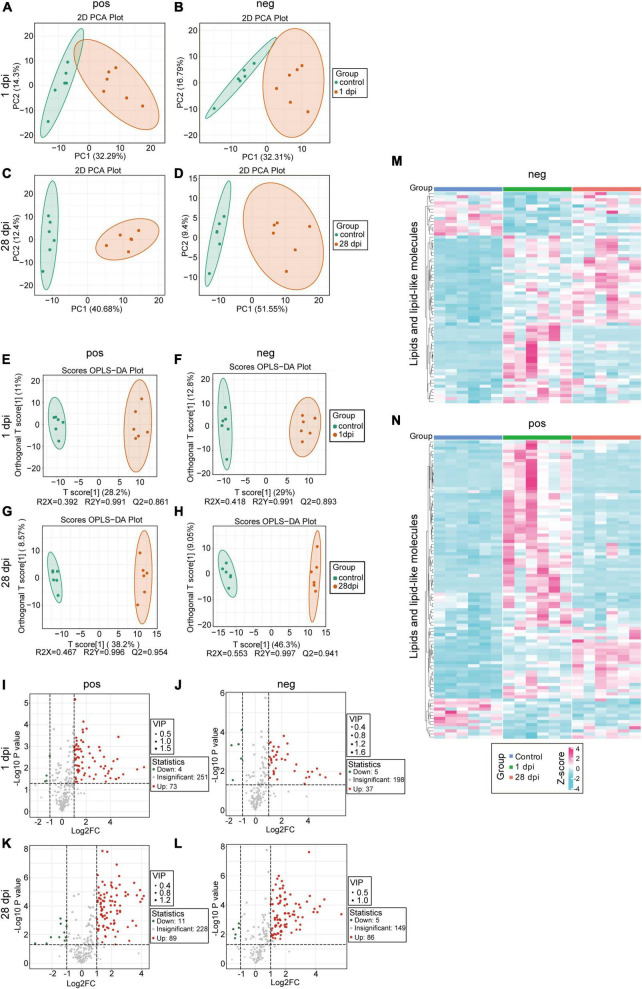
Impaired local lipid metabolism after spinal cord injury (SCI). **(A,B)** Principal component analysis (PCA) of local lipid metabolites in the injured spinal cord tissues, comparing the SCI groups at 1 dpi (orange) to the control groups (green). **(C,D)** PCA of local lipid metabolites in the injured spinal cord tissues, comparing the SCI groups at 28 dpi (orange) with the control groups (green). **(E,F)** Orthogonal partial least squares-discriminant analysis (OPLS-DA) of local lipid metabolites in the injured spinal cord tissues, comparing the SCI groups at 1 dpi (orange) with the control groups (green). **(G,H)** OPLS-DA of local lipid metabolites in the injured spinal cord tissues, comparing the SCI groups at 28 dpi (orange) with the control groups (green). **(I–L)** Volcano plot comparing the SCI groups at 1 dpi **(I,J)** and 28 dpi **(K,L)** with the control groups; fold change (FC) < 1.5 and *p* < 0.05. **(M,N)** Heatmaps comparing the SCI groups at 1 and 28 dpi with the control groups; *p* < 0.05, VIP > 1, and absolute value of FC > 1.5. *n* = 6 mice per group. “Neg” represents anion detection mode, while “pos” represents cation detection mode.

To elucidate the lipid metabolism status at the lesion site, metabolic sequences from the spinal cord tissues collected from control and SCI mice at 1 (acute-phase) and 28 (chronic-phase) dpi were compared. Lipid metabolism-related products were selected from the acquired metabolomic data and subjected to a series of bioinformatics analyses. PCA and OPLS-DA revealed a significant alteration in the distribution of multiple lipid metabolism components, which persisted into the chronic phase (Figures 1A–H), suggesting a pronounced disruption of lipid metabolism in the injured spinal cord. Volcano plot analysis revealed that the majority of lipid metabolism-related products exhibited early and sustained increases in the SCI tissues compared to controls (Figures 1I–L), indicating prolonged lipid microenvironment dysregulation in the injured spinal cord after SCI. Upon comparing lipid metabolism differences between the control, acute-phase SCI (1 dpi), and chronic-phase SCI (28 dpi) groups, dynamic changes in the deposition of long-term lipid metabolism products, rather than sustained elevation of specific lipid classes, were observed, as evidenced by the heatmap results (Figures 1M, N). Those findings suggest that changes in lipid metabolism are characterized by dynamic and prolonged imbalances, and clearly demonstrates the sustained deposition of lipid metabolism products in the injured spinal cord.

**FIGURE 2:**
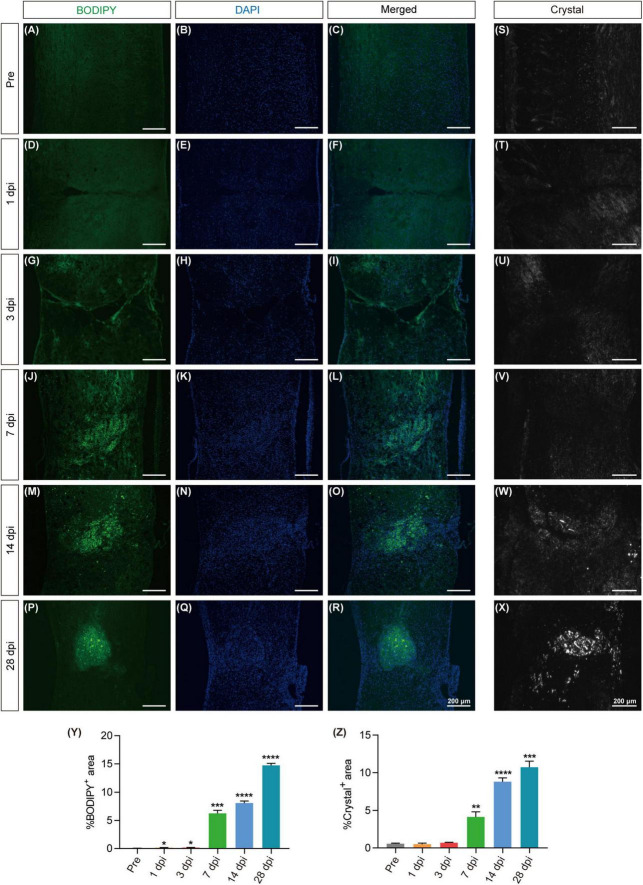
Excessive lipid droplet and cholesterol crystal formation in the injured area after SCI. **(A–R)** Fluorescence images of BODIPY within the spinal cord tissues, depicting the formation of lipid droplets (green) in the injured area before injury (pre) and at 1, 3, 7, 14, and 28 dpi. DAPI staining was used for cell nuclei (blue). **(S–X)** Polarized light detection images reveal the presence of cholesterol crystals in the injured area at pre, 3, 7, 14, and 28 dpi. **(Y,Z)** Quantitative analysis of the percentage of BODIPY^+^ **(A–R)** or Crystal^+^ **(S–X)** in the injured area at pre, 1, 3, 7, 14, and 28 dpi. *n* = 3 mice per group for immunofluorescence analysis and polarized light detection. *p < 0.05, ***p < 0.001 and ****p < 0.0001 (1, 3, 7, 14, and 28 dpi vs. pre of the proportion of BODIPY^+^ in the injured area). **p < 0.01, ***p < 0.001 and ****p < 0.0001 (1, 3, 7, 14, and 28 dpi vs. pre of the proportion of Crystal^+^ in the injured area).

To further clarify the spatiotemporal variations in lipid accumulation at the lesion site, we employed BODIPY staining and polarization microscopy on frozen sections of the spinal cord at pre, 1, 3, 7, 14 and 28 dpi. BODIPY staining revealed a notable accumulation of lipid droplets at 7 dpi, characterized by small volumes and widespread distribution, both of which continued to increase over time. On 28 dpi, lipid droplets aggregates were observed around the lesion epicenter, with larger volumes and a clustered distribution patterns (Figures 2A–R, Y). The appearance of cholesterol crystals at the lesion site was observed on 14 dpi using polarized light imaging technology, with a subsequent increase in both quantity and size by 28 dpi (Figures 2S–X, Z). These results suggest a persistent lipid droplet and cholesterol crystal accumulation at the lesion site, indicating a failure of metabolic clearance.

### 3.2 Increased pu.1 expression at the lesion site after SCI

**FIGURE 3:**
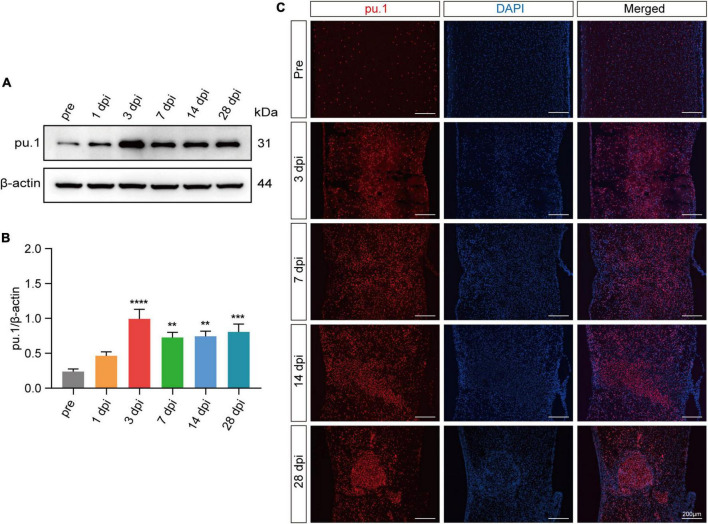
Expression of pu.1 after SCI. **(A)** Western blot analysis of pu.1 protein expression in the control and injured spinal cord tissues of mice. **(B)** Quantitative analysis of pu.1 protein expression at different time points in panel **(A)**. **(C)** Immunofluorescence staining showing the expression of pu.1 protein (red) within the spinal cord tissues at pre, 3, 7, 14, and 28 dpi. DAPI staining was used for cell nuclei (blue). *n* = 3 mice per group for western blot assay and immunofluorescence analysis. **p < 0.01, ***p < 0.001 and ****p < 0.0001 (1, 3, 7, 14, and 28dpi vs. pre of pu.1\β-actin).

As pu.1 has emerged as a pivotal regulator in metabolic diseases (Lackey et al., 2019; Liu et al., 2020; Pimenova et al., 2021), its specific role in governing lipid metabolism after SCI remains elusive. To address this gap, Western blot and immunostaining were used to detect pu.1 expression at the lesion site after SCI. Western blot analysis revealed a progressive increase in pu.1 protein expression, beginning at 1 dpi, peaking at 3 dpi, and maintaining elevated levels over an extended period (Figures 3A, B). The immunofluorescence results further supported these findings (Figure 3C), indicating extensive activation and prolonged high pu.1 expression at the lesion site after SCI. This finding underscores the potential functional significance of pu.1 in the pathological processes associated with SCI.

### 3.3 Pu.1 is mainly expressed in macrophages/microglia after SCI

**FIGURE 4:**
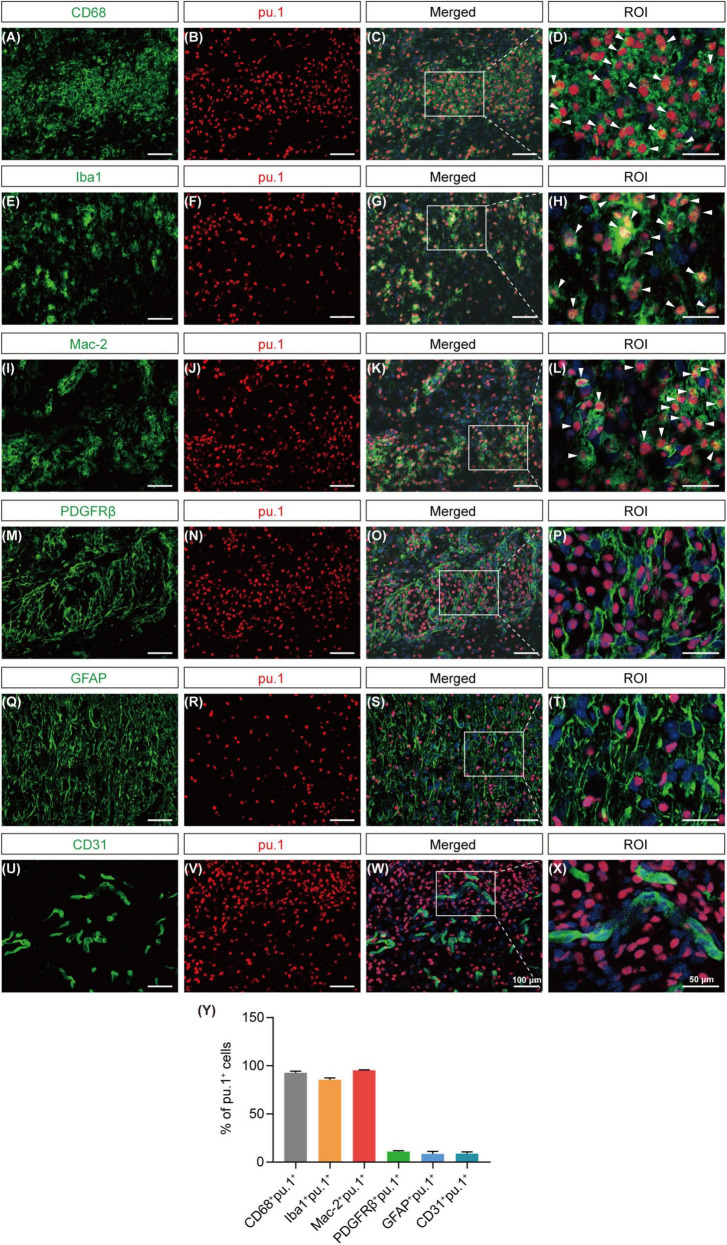
Cellular localization of pu.1 expression in the injured area after SCI. **(A–D)** Immunofluorescence staining showing the co-localization of pu.1 (red) and microglia/macrophages (CD68; green) in the injured area at 14 dpi. **(E–H)** Immunofluorescence staining showing the co-localization of pu.1 (red) and microglia/macrophages (Iba1; green) in the injured area at 14 dpi. **(I–L)** Immunofluorescence staining showing the co-localization of pu.1 (red) and foamy macrophages (Mac-2; green) in the injured area at 14 dpi. **(M–P)** Immunofluorescence staining of pu.1 (red) and fibroblasts (PDGFRβ; green) in the injured area at 14 dpi. **(Q–T)** Immunofluorescence staining of pu.1 (red) and astrocytes (GFAP; green) in the perilesional area at 14 dpi. **(U–X)** Immunofluorescence staining of pu.1 (red) and vascular endothelial cells (CD31; green) in the injured area at 14 dpi. The region of interest (ROI) represents the boxed region on the left. Arrowheads indicate CD68^+^pu.1^+^ **(A–D)**, Iba1^+^pu.1^+^ **(E–H)**, or Mac-2^+^pu.1^+^ **(I–L)** cells at 14 dpi. **(Y)** Quantification of the proportion of CD68^+^pu.1^+^, Iba1^+^pu.1^+^, Mac-2^+^pu.1^+^, PDGFRβ^+^pu.1^+^, GFAP^+^pu.1^+^, and CD31^+^pu.1^+^ cells at 14 dpi. *n* = 3 mice per group for immunofluorescence analysis.

To preliminarily identify the cellular sources of pu.1, we conducted co-staining with key cell components of the lesion site, including CD68^+^/Iba1^+^ cells (activated macrophages or microglia), Mac-2^+^ cells (macrophages), PDGFRβ^+^ cells (fibroblasts), GFAP^+^ cells (astrocytes), and CD31^+^ cells (vascular endothelial cells). At 14 dpi, pu.1 was mainly located in CD68^+^, Iba1^+^, and Mac-2^+^ cells (Figures 4A–L, Y). Notably, no significant colocalization was observed between pu.1 and GFAP^+^ astrocytes, PDGFRβ^+^ fibroblasts, or CD31^+^ vascular endothelial cells (Figures 4M–Y), suggesting that pu.1 was predominantly produced within macrophages/microglia after SCI.

### 3.4 DB1976 treatment reduces lipid droplet aggregation and cholesterol crystal formation after SCI

**FIGURE 5:**
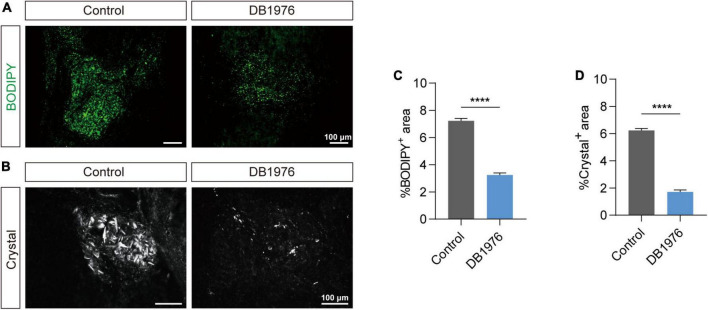
DB1976 treatment reduces lipid droplet accumulation and cholesterol crystal formation in the injured spinal cord. **(A)** BODIPY staining of lipid droplets in the sagittal sections at 28 dpi in the control and DB1976 groups. **(B)** Polarized light detection of cholesterol crystals in the sagittal sections at 28 dpi in the control and DB1976 groups. **(C,D)** Quantitative analysis of the percentage of BODIPY^+^ **(A)** or Crystal^+^ **(B)** in the injured area of the control and DB1976 groups. *n* = 3 mice per group for immunofluorescence analysis and polarized light detection. ****p < 0.0001.

Since suppression of pu.1 in microglial cells has been shown to elevate the expression of genes associated with lipid metabolism and activate related pathways (Pimenova et al., 2021), we hypothesized that inhibiting pu.1 function could potentially ameliorate lipid deposition and cholesterol crystal formation after SCI. DB1976, a heterocyclic diamine that competitively inhibits the binding of pu.1 to DNA, exhibits minimal impact on other ETS transcription factors and a superior safety profile without compromising cell viability (Antony-Debré et al., 2017). To minimize the potential systemic effects of DB1976, we opted for local intrathecal administration. Lipid staining and cholesterol crystal examination at the lesion site at 28 dpi revealed that DB1976 treatment significantly reduced lipid droplet aggregation and cholesterol crystal formation at the lesion site (*p* < 0.0001) (Figure 5).

### 3.5 DB1976 treatment reduces the inflammatory response and promotes tight junction restoration between vascular endothelial cells after SCI

**FIGURE 6:**
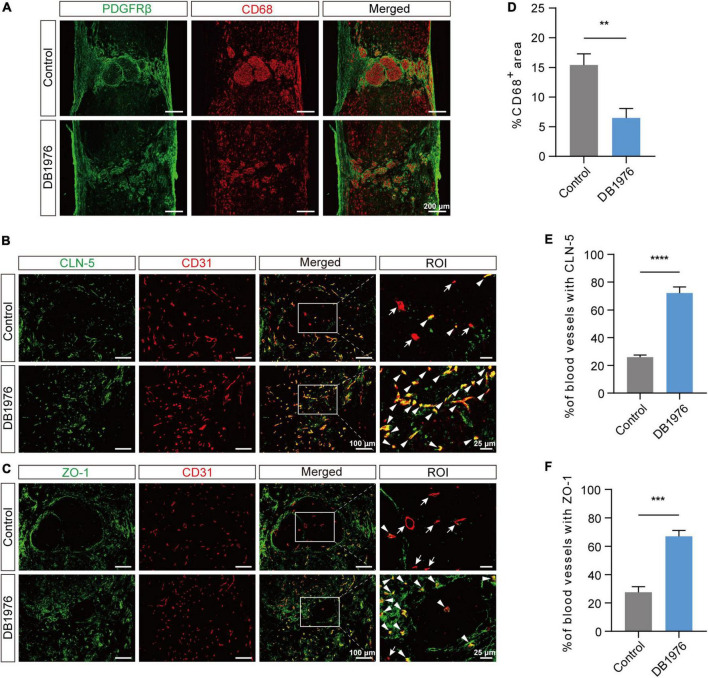
DB1976 treatment reduces chronic inflammatory response and promotes the repair of tight junctions between endothelial cells in the injured area after SCI. **(A)** Immunofluorescence staining of CD68 (red) and PDGFRβ (green) in the sagittal sections at 28 dpi in the control and DB1976 groups. **(B,C)** Immunofluorescence staining of CD31 (red; blood vessels) and **(B)** CLN-5 (green; tight junction protein) or **(C)** ZO-1 (green; tight junction protein) in the sagittal sections at 28 dpi in the control and DB1976 groups. ROI represents the boxed region on the left. Arrowheads indicate CLN-5^+^ **(B)** or ZO-1^+^ **(C)** vessels, while arrows indicate CLN-5^–^ **(B)** or ZO-1^–^ **(C)** vessels. **(D)** Quantitative analysis of the percentage of CD68^+^ area in the sagittal sections at 28 dpi in the control and DB1976 groups. **(E,F)** Quantitative analysis of the percentage of CLN-5^+^ **(B)** or ZO-1^+^ **(C)** vessels in the lesion epicenter of the control and DB1976 groups at 28 dpi. *n* = 3 mice per group for immunofluorescence analysis. **p < 0.01, ***p < 0.001, ****p < 0.0001.

After SCI, macrophages gather at the lesion epicenter and adopt a pro-inflammatory foamy cell phenotype, largely due to the excessive uptake of lipid debris (Ren and Young, 2013; Wang et al., 2015; Ryan et al., 2022). To assess whether DB1976 treatment can alleviate the persistent inflammatory response at the lesion site after SCI, we conducted CD68 staining at 28 dpi. The results revealed a notable reduction in the area occupied by CD68^+^ inflammatory cells within the injured spinal cord of SCI mice after DB1976 treatment (Figures 6A, D), suggesting that targeting pu.1 with DB1976 helps alleviate inflammation during the chronic phase after SCI.

Given that foamy macrophages disrupt tight junctions (TJs), inhibit the reconstruction of BSCB, and aggravate secondary injury (Wang et al., 2015; Luo et al., 2022), we examined the impact of DB1976 treatment on TJs. Compared to the control group, DB1976 treatment significantly increased TJ formation, as evidenced by positive staining for CLN-5 and ZO-1 within CD31^+^ vessels (Figures 6B, C, E, F). This suggests that DB1976 treatment significantly enhances TJ formation in regenerating vessels, thereby promoting BSCB establishment.

### 3.6 DB1976 treatment reduces fibrotic scar formation and promotes glial connectivity in the injured area after SCI

**FIGURE 7:**
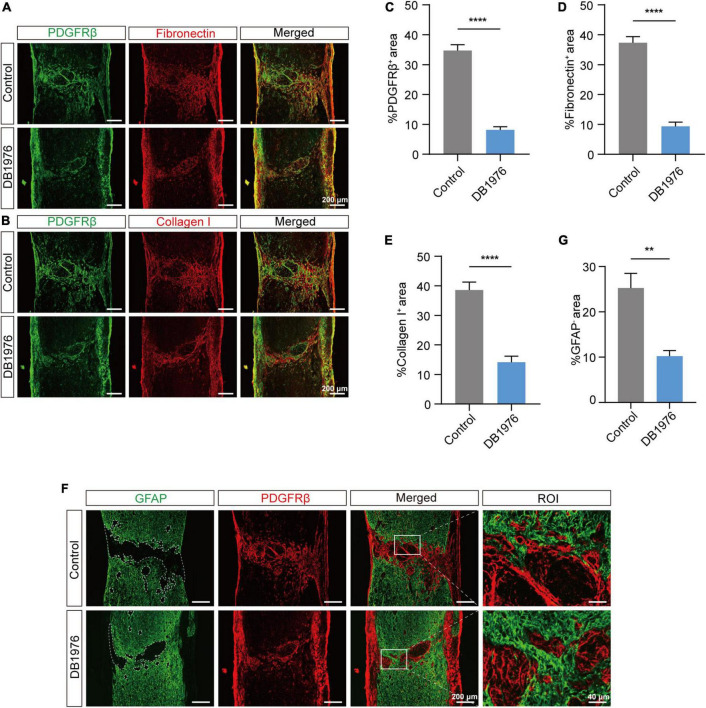
DB1976 treatment reduces fibrotic scar formation and promotes glial connectivity in the injured area after SCI. **(A)** Immunofluorescence staining of fibronectin (red) and PDGFRβ (green) in the sagittal sections at 28 dpi in the control and DB1976 groups. **(B)** Immunofluorescence staining of Collagen I (red) and PDGFRβ (green) in the sagittal sections at 28 dpi in the control and DB1976 groups. **(C–E)** Quantitative analysis of the percentage of PDGFRβ^+^, Fibronectin^+^, and Collagen I^+^ areas at the injury area in the control and DB1976 groups. **(F)** Immunofluorescence staining of GFAP (green) and PDGFRβ (red) in the sagittal sections at 28 dpi in the control and DB1976 groups. ROI represents the boxed region on the left. **(G)** Quantitative analysis of the area of the lesion epicenter (GFAP^–^) in the control and DB1976 groups. *n* = 3 mice per group for immunofluorescence analysis. **p < 0.01, ****p < 0.0001.

To confirm the effect of DB1976 treatment on fibrotic and astrocytic scarring, we performed immunofluorescence staining at 28 dpi. Our results demonstrated a significant reduction in the fibrotic scar area within the lesion site of the DB1976 treatment group, with notable decreases in the PDGFRβ^+^ (Figures 7A, C), Collagen I^+^ (Figures 7B, E), and Fibronectin^+^ (Figures 7A, D) areas. Additionally, the GFAP^+^ astrocytic scar in the injured area of the DB1976 treatment group extended across the lesion epicenter, connecting the rostral and caudal sides of the lesion site (Figures 7F, G). The GFAP^–^ area of the lesion was also significantly reduced in the DB1976 treatment group (Figures 7F, G). These findings indicate that DB1976 treatment significantly diminishes fibrotic scar formation, promotes wound healing, and facilitates the establishment of glial connections within the injured area.

## 4 Discussion

This study revealed that the strategic inhibition of pu.1 using the small molecule antagonist DB1976 confers multiple therapeutic benefits, including reduced lipid droplet deposition, cholesterol crystal formation, inflammation, fibrotic scarring and enhanced glial bridging at the lesion site and TJ formation within blood vessels. Together, these findings suggest that targeting pu.1 represents a promising therapeutic strategy for SCI, with the potential to improve pathological outcomes and address repair failure.

The spinal cord releases substantial lipid-rich myelin and cellular debris after injury, resulting in a region burdened with a high lipid load during the debris clearance phase (Wang et al., 2015; Ryan et al., 2022). However, the specific spatiotemporal dynamics of lipid metabolism in the local tissue post-SCI remain relatively unexplored. Our study revealed a rapid, profound, and enduring dysregulation of lipid metabolism post-SCI, which hindered the timely clearance of lipids, leading to the accumulation of lipid droplets and subsequent cholesterol crystal formation at the lesion site. These needle-like cholesterol crystals emerge when tissues fail to efficiently metabolize excess cholesterol. Notably, cholesterol depletion has been shown to promote axon growth *in vitro* and peripheral nerve regeneration *in vivo* (Roselló-Busquets et al., 2019). The inhibition of cholesterol biosynthesis through molecular and pharmacological interventions has proven effective in promoting survival and regeneration in the central nervous system, particularly in retinal ganglion cells after optic nerve injury (Shabanzadeh et al., 2021). Studies involving myelin basic protein-deficient shiverer mice (Chernoff, 1981) have demonstrated the inhibitory role of myelin lipids in the central nervous system, especially cholesterol and sphingomyelin, on axon regeneration after SCI (Mar et al., 2016). Furthermore, investigations in shiverer mice and lipid scavenging at the injury site have demonstrated a significant increase in dorsal column sensory axon regeneration after SCI (Mar et al., 2016). Collectively, these findings underscore the therapeutic potential of modulating lipid metabolism, particularly cholesterol biosynthesis, for promoting axon regeneration in both the central and peripheral nervous systems.

Recently, the transcription factor pu.1 has been recognized as a pivotal regulator in metabolic disorders (Lackey et al., 2019; Liu et al., 2020; Pimenova et al., 2021), prompting investigations into its potential role in governing lipid metabolism alterations after SCI. Nonetheless, the spatiotemporal expression of pu.1 and its impact on lipid metabolism after SCI remain unclear. Our study identified a rapid upregulation of pu.1 expression in the acute phase post-injury, persisting for at least 28 dpi, with a marked presence in macrophages/microglia. In the context of SCI, macrophages/microglia are primary mediators of myelin debris clearance through phagocytosis (Greenhalgh and David, 2014; Van Broeckhoven et al., 2021; St-Pierre et al., 2023). The infiltration of macrophages into the SCI lesion to clear lipid-rich debris gives rise to foamy macrophages within the lesion epicenter. However, their decreased efficiency in processing phagocytosed material leads to a persistent inflammatory reaction in the injured area. Previous studies have highlighted the transcriptional profiles of these lipid-laden foamy macrophages at 7 dpi, revealing a predominance of lipid catabolism based on gene ontology analysis (Zhu et al., 2017). This timeframe aligns with a crucial period for scar formation. Moreover, the spinal cord in neonatal P2 mice lacks myelin-derived lipids and can undergo scar-free repair after injury (Li et al., 2020). These findings suggest a potential interplay between foam cell generation and scar formation in the injury area.

We used DB1976 to determine whether pu.1 regulates the pathological shifts in lipid metabolism within these cells after SCI. DB1976 is a small-molecule selenium-thiophene analog of DB270 that effectively inhibits pu.1 by penetrating cells and interfering with its DNA binding capacity, rather than directly binding to the protein (Munde et al., 2014). Due to its specificity for the AT-enriched wing sequence within the pu.1 binding domain, DB1976 selectively modulates pu.1 activity without affecting other E26 transcription factors. With a relatively high half-maximal inhibitory concentration (IC50, 105 μM vs. <8 μM), DB1976 ensures enhanced safety and minimal impact on cell viability (Antony-Debré et al., 2017). Upon comparing outcomes between the DB1976 treatment and control groups, we observed a significant reduction in the deposition of lipid droplets and cholesterol crystals within the lesion site following pu.1 inhibition. Additionally, a notable reduction in fibrotic scar formation and chronic inflammatory cell aggregation was observed, as well as a marked increased glial bridging promotion and TJ formation within blood vessels in the injury area. While glial scars have been traditionally viewed as barriers to axonal regrowth, recent evidence suggests a beneficial role in tissue repair and axon regeneration (Rolls et al., 2009; Anderson et al., 2016; Yang et al., 2020). Conversely, fibrotic scars, primarily composed of fibroblasts, are known to impede axon regeneration (Soderblom et al., 2013; Dias et al., 2018). Unlike the sustained accumulation of activated macrophages/microglia in adult lesions, transient microglial activation mediates glial bridge formation and organizes scar-free healing in neonatal mice (Li et al., 2020), similar to the regenerative processes observed in zebrafish and newts (Zukor et al., 2011; Mokalled et al., 2016). Our previous study has also demonstrated that restoring BSCB function using tocilizumab can promote axon regeneration and motor function recovery after SCI (Luo et al., 2023). The present study indicates that targeting pu.1 using DB1976 treatment can reduce the formation of fibrotic scars in the injured area, promote glial connections, dampen inflammatory reactions, and enhance BSCB integrity.

Nonetheless, further studies are needed to elucidate the regulatory mechanisms and effects on neuronal apoptosis and astrocyte activation under different lipid states. In vitro studies with co-cultured macrophages and astrocytes or neurons under different lipid conditions may be particularly useful. Investigating the effects of DB1976 on neural retention, regeneration, and functional recovery in mice is also essential. Additionally, more comprehensive investigations exploring the pathological changes in specific products and pathways related to local lipid metabolism, along with the precise effects of pu.1 on lipid metabolism in macrophages and microglia at the single-cell level, are warranted.

## 5 Conclusion

In conclusion, targeting pu.1 was found to rescue lipid metabolism dysregulation and ameliorate the pathological progression following SCI. Thus, we propose pu.1 as a possible molecular target for preventing scar formation. Furthermore, we have shown that pu.1 inhibition prevents inflammation enlargement, thus protecting BSCB disruption. Overall, these findings enhance our understanding of the mechanisms underlying the failure of repair following SCI.

## Data availability statement

The datasets presented in this study can be found in online repositories. The names of the repository/repositories and accession number(s) can be found at: MetaboLights (www.ebi.ac.uk/metabolights/MTBLS10453), Project accession: MTBLS10453.

## Ethics statement

The animal study was approved by the Animal Ethics Committee of Anhui Medical University (Approval No. LLSC20160052). The study was conducted in accordance with the local legislation and institutional requirements.

## Author contributions

YS: Writing – original draft, Software, Methodology, Formal analysis, Data curation, Conceptualization. MZ: Writing – review and editing, Methodology, Funding acquisition, Formal analysis, Conceptualization. YL: Writing – original draft, Software, Methodology, Formal analysis, Data curation, Conceptualization. JL: Writing – original draft, Validation, Software, Resources. FO: Writing – original draft, Validation, Software, Resources. YZ: Writing – original draft, Methodology, Data curation. JW: Writing – original draft, Methodology, Data curation. ZM: Writing – original draft, Validation, Software. CM: Writing – original draft, Validation, Software. YB: Writing – review and editing, Supervision, Project administration, Funding acquisition. LC: Writing – review and editing, Supervision, Project administration, Funding acquisition. JJ: Writing – review and editing, Supervision, Project administration, Funding acquisition.

## Footnotes

1
https://metlin.scripps.edu/

2
https://hmdb.ca/

3
https://www.kegg.jp/

4
https://mona.fiehnlab.ucdavis.edu/

5
http://www.massbank.jp/
